# Supplementary material for: Initial assessment of the spatial learning, reversal, and sequencing task capabilities of knock-in rats with humanizing mutations in the Aβ-coding region of App
Source: PLoS One. 2022 May 4;17(5):e0263546. doi: 10.1371/journal.pone.0263546 (PMC9067689; doi:10.1371/journal.pone.0263546)
Supplement: Attachment — Submitted filename: response to the revision of the revision.docx [file pone.0263546.s002.docx]

Q: Corner rank: The interpretation of a corner task as a measure of social dominance, although it is very interesting data, is not supported by data on social hierarchy. It should be discussed as a limitation of the study.

R: We have eliminated any reference to this test as a measure of social dominance. The methods now read

*“Corner rank comparison.* To understand better the effect of social interaction on the behavior of animals in the IntelliCage, we ranked animals via a point system based on whether an animal visits the correct corner more than other animals do during single corner restriction. This may occur with the exclusion of other animals from those corners, which may bear upon the performance of paired animals in subsequent tests.”

Q: Activity curves: The name of this variable (activity curves) is misleading as this variable measures the fraction of correct responses (relative to visits to the opposite corner) rather than "activity", a word that strongly associates with motor/exploratory activity. The choice of this measure (proportion of correct to opposite corner) to characterize cognitive performance is not clearly explained. Why the visits to two other corners were excluded is not explained.

R: We have changed the name activity curves into Learning curves.

As for measuring the correct versus opposite, we realize that the writing in the methods was confusing. The sections describing the methods and rationale now read:

*“Learning curves.* To visualize learning of all the animals in each IntelliCage as a unit, we charted the fractional accumulation of correct visits (also opposite visits for the Behavioral sequencing and Serial reversal tasks) over the course of each drinking session. With the resulting curves, we can qualitatively compare task performance according to drinking session, sex, and Run. We followed this workflow to produce the learning curves for each program: 1. For each subset of rats by sex, cohort, and Run (e.g., cohort A males in their Run-1), count the total number of visits those rats made for each drinking session. For a 3-day program, there should be 3 totals for a given subset. 2. For each subset as described in step 1, tabulate the fractional accumulation of correct (and opposite) visits over time for each drinking session, adding to each fraction, starting from 0, the value of 1 divided by the associated total count for that subset and drinking session each time a correct (or opposite) visit occurs, and 0 otherwise.”

*“Statistical analysis of area under the curves (AUC).* To assess task performance quantitatively, we used the area under the learning curves of individual animals in each IntelliCage. Every correct visit during a drinking session contributes to this area; this approach accounts not only for the total fraction of correct visits but also for the rate at which they accumulate. It also takes advantage of the large volume of data the IntelliCage collects from each program such that there is no need to approximate the rate of learning with curve fitting. For the Behavioral sequencing and the Serial reversal tasks we also calculated the AUC for the opposite corner, since the opposite corner represents the previously correct corner. Thus, calculations of these two areas indicates the speed by which a rat learns the rules regulating alternation of correct to previously correct corners.”

As for the analysis of all for corners (i.e. the incorrect corners), reviewer 2 pointed out during the first round of revision, the following:

*If " activity curves show.. the fractional accumulation of corner visits" as stated in Results, then the results for correct and incorrect activity curves are dependent on each other and do not need to be represented as separate outcomes.*

Based on this comment we eliminated the analysis of the other corners when revising the manuscript.

Q: The analyses of reversal learning would benefit from analyses of correct vs previously baited corners.

R: Thank you for the suggestion but this analysis, which is conceptually identical to the analysis of Opposite in Behavioral sequencing and Serial reversal, is not informative in this case.

Q: The idea that "Apph/h rats constitute control animals" should be proven by data showing comparison of this model to wt rats. For example, we do know that presence of wild type human tau in mice does have consequences on a number of biological levels. This should be discussed as a limitation/future direction of the study.
R: We have added this sentence to the introduction.

“Whether expression of human Aβ is, per se’, sufficient to impact behavior will be addressed in future studies comparing *App^h/h^* to *App^w/w^* rats.”

Q: Discussion section is much improved. A particular discussion on chance levels in different tasks is a particularly important addition. I would suggest introducing definitions of chance level for appropriate variables (including AUC) in the Method section, and, what would be really great, putting these levels as a line in appropriate figures. The issue of chance levels is especially significant for this study as its goal is to prove that rats learned the tasks.

R: Thank you for the suggestion but we think that the chance level is sufficiently explained in the discussion and very intuitive for a system with 4 options.

Q: In the discussion, it is stated that "...a program of intermediate difficulty using a sequence involving all four corners (in clockwise motion, for example) rather than just two in a single diagonal, might be worth testing in future studies." Although the main idea here (that a task of intermediate difficulty would be the most useful) sounds great, a particular example (visiting all four corners in clockwise motion) might be easily affected by development of pervasive strategy (to visit each next corner) that can collapse the cognitive demand of this particular protocol.
R: We have added this sentence to the discussion.

“Yet, such a program could be affected by development of pervasive strategy (to visit each next corner) that can collapse the cognitive demand of this particular protocol.”

Minor:
Q: - abbreviations should be explained before their first use - a KI approach in mice...

R: We have added the KI abbreviation in the Abstract

.
Q: - a statement in the Introduction that " ...The KI approach ... makes no assumption about pathogenic mechanisms (except the unbiased genetic one)" is rather unclear and not needed.

R: The sentence has been deleted

Q: - The figures are improved; but there is still a lot of information that is unreasonably hidden. For example, if FP = 1st pass, SP = 2nd pass, the figures would be much more readable if "Run 1" and "Run 2" would be used instead of FP and SP. The same could be said about other abbreviations (in the figures and Tables) which could be easily avoided altogether (BS-O, BS-C, SR, ...). While these comments may sound like non-essential for investigators who worked with these tasks, the relative novelty of ItelliCage, tasks and variables to overwhelming majority of other researchers makes it very important to spent time and make data presentation as clear as possible.

R: First pass and second pass have need changed to Run-1 and Run-2 in text and figure.

Abbreviations have been eliminated.
